# High-Fat Diet Anticipates Age-Related Sarcopenia Through Increased Oxidative Stress and Inflammation

**DOI:** 10.3389/bjbs.2026.15743

**Published:** 2026-02-26

**Authors:** Fabiano Cimmino, Lidia Petrella, Gina Cavaliere, Mariarosaria Negri, Claudia Pivonello, Giuliana Napolitano, Marianna Crispino, Giovanna Trinchese, Annamaria Colao, Maria Pina Mollica

**Affiliations:** 1 Department of Biology, University of Naples Federico II, Naples, Italy; 2 Department of Pharmaceutical Sciences, University of Perugia, Perugia, Italy; 3 Dipartimento di Benessere, Nutrizione e Sport, Università Telematica Pegaso, Naples, Italy; 4 Department of Public Health, University of Naples Federico II, Naples, Italy; 5 UNESCO Chair for Health Education and Sustainable Development, University of Naples Federico II, Naples, Italy

**Keywords:** aging, body fat mass, body lean mass, inflammation, obesity

## Abstract

**Background:**

Ageing, a physiological process, and obesity, a pathological condition, are both associated with several metabolic alterations including energy imbalance, altered body composition, chronic low-grade inflammation, lipotoxicity, glucotoxicity, insulin resistance and mitochondrial dysfunctions. During ageing mitochondrial capacity declines and oxidative stress increases. However, the biphasic model of age-associated mitochondrial functions indicates that, before the ageing-associated decrease in mitochondrial respiration, this parameter increases in the transition from young adult to middle-aged, with a concomitant mild increase in ROS production that stimulates an antioxidant response, limiting the ageing-associated damages. Ageing-associated body composition changes can lead to sarcopenia, one of the most debilitating dysfunctions in the elderly. The sarcopenia is a known geriatric syndrome characterized by the loss of muscle mass and strength and mitochondria dysfunctions. These alterations of the disease can be exacerbated by obesity. Here, in an experimental animal model of diet-induced obesity, we evaluated the time-course changes in body composition, inflammatory and oxidative stress parameters, mitochondrial functions and antioxidant responses.

**Methods:**

Male Wistar rats at 60 days of age were divided into two experimental groups: the first group received a standard diet; the second group received a high-fat diet (HFD). The animals from both groups were fed with the appropriate diet for 1, 3, 6, 12, or 24 weeks (n = 6 for each group and time point). At each time point, the animals were sacrificed and dissected to obtain the organs and tissues needed for analysis.

**Results:**

Our results clearly showed the contribution of high-fat diet in anticipating and worsening the metabolic and inflammatory alterations associated with age, in particular, highlighting the role of mitochondria in attempting the regulation of physiological alterations typical of aging.

**Conclusion:**

In the HFD group the antioxidant defences fail their job because of the additional inflammation and oxidative stress due to the diet. HFD is related to decreased animals’ activity. Thus, cannot be excluded that the reduced physical activity may contribute, at least in part, to the impaired mitochondrial functions in the skeletal muscle of HFD rats. Altogether, our results clearly highlighted the contribution of HFD in anticipating and worsening the metabolic and inflammatory alterations associated with aging, including sarcopenia.

## Graphical Abstract

**Figure:**
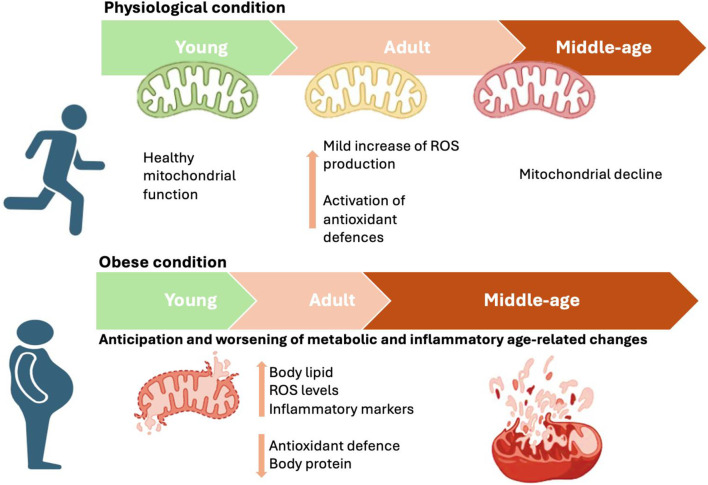
In the transition from youth to adult of healthy individuals, the physiological increase of mitochondrial activity paralleles to a mild increase in ROS production that stimulates the antioxidant response limiting the aging-associated damages. Obesity leads to mitochondrial dysfunction and oxidative stress, anticipating and worsening the metabolic and inflammatory alterations associated with age.

## Introduction

Aging is associated with changes in body composition with an increase in fat mass and a decrease in lean mass percentage [1]. Similar changes are observed also in obesity condition, a chronic disease characterized by an excessive accumulation of adipose tissue producing cytokines and factors responsible for the chronic low-grade inflammatory state and oxidative stress [2–6]. Obesity is mainly due to an imbalance between energy consumption and metabolizable energy intake [7]. Also during the aging process, this balance is altered, due to the progressive malfunction of mechanisms controlling energy homeostasis [8].

An increasing amount of data indicates a correlation between aging, obesity and sarcopenia [9]. Sarcopenia is a pathological condition affecting skeletal muscle and characterized by loss of muscle strength but also a decrease of muscle quantity and quality. These features affect physical performance contributing to increased risk of bone fractures, worsening the quality of life [10, 11]. The prevalence of obesity combined with sarcopenia has been increasing in older adults, especially in industrialized countries [12]. The simultaneous presence of sarcopenia (loss of muscle mass and function) and adiposity (increase in fat mass) identifies a clinical and functional condition termed sarcopenic obesity (SO) [13]. Many factors are associated with SO such as inflammatory and immunological factors, and sex-specific hormonal changes [14, 15]. SO dramatically increases mortality and morbidity rates in older adults in whom the reduction in lean mass parallels with the increase in fat mass leading to metabolic alterations [16–18].

With advancing age, there is a progressive decline in mitochondrial capacity accompanied by a sustained increase in oxidative stress and ROS production [19, 20]. In this context, it is important to consider the biphasic model of age-associated mitochondrial dysregulation, indicating that mitochondrial capacity increases from young adult to middle-aged and subsequently decreases in later stages of life [21]. The adult-associated increased mitochondrial activity is linked with mild ROS production, representing a physiological adaptation mechanism [21, 22]. Indeed, this mild ROS production can stimulate an antioxidant response that limits the aging-associated damages and therefore promotes a healthy transition from adulthood to aging [21, 23].

However, how this biphasic mitochondrial trajectory is affected by chronic metabolic stressors such as high-fat diet (HFD)-induced obesity, and how it is temporally linked with the development of inflammation, muscle dysfunction, and sarcopenia, remains poorly understood.

In the present study, we employed a longitudinal experimental design in a rodent model of high-fat diet (HFD)-induced obesity, to systematically investigate the time-dependent interplay between inflammation, body composition remodeling, skeletal muscle mitochondrial functions, and antioxidant responses across aging. This approach, by integrating the biphasic model of mitochondrial function with longitudinal metabolic and functional outcomes, provides novel insights into the temporal mechanisms linking chronic inflammation, mitochondrial dysregulation, and the progression toward sarcopenic obesity. The findings of this study help to clarify the temporal sequence through which aging and high fat diet synergistically drive muscle dysfunctions.

## Materials and Methods

### Materials Reagent

All chemicals were purchased from Sigma-Aldrich (St. Louis, MO, USA), unless otherwise specified.

### Animal Diet

Male Wistar rats at 60 days of age and 345.7 g of average body weight were individually caged in a temperature-controlled room and exposed to a daily 12/12 h light/dark cycle with free access to diet and drinking water. Rats were divided into two experimental groups according to a different dietary regimen: the first group (control CD) received a standard diet (15.88 kJ/g; 10.6% fat J/J, 29% protein J/J, 60.4% carbohydrate J/J); the second group (HFD) received high-fat diet (22.1 kJ/g; 40% fat J/J, 29% protein J/J, 31% carbohydrate J/J) [24–27]. The animals from both groups were fed with the appropriate diet for 1, 3, 6, 12, or 24 weeks (n = 6 for each group and time point). An additional group (n = 6) was sacrificed at the beginning of the study to establish baseline measurements of body compositions. At the end of each time point, the rats were anesthetized, decapitated with a guillotine, and the blood was taken from the inferior cava vein. Serum or plasma was obtained by centrifugation at 1,000 × g for 10 min at 4 °C. The skeletal muscle was quickly excised, an aliquot was used for the mitochondrial isolation, the remaining was stored at −80 °C for further measurements. Procedures involving animals and their care were conducted in conformity with international and national laws and policies (EU Directive2010/63/EU for animal experiments, ARRIVE guidelines, and the Basel declaration including the 3R concept). The procedures reported here were approved by the Institutional Committee on the Ethics of Animal Experiments (CSV) of the University of Naples Federico II (Permit Number: 2010/0149862) and by the “Ministero della Salute”.

### Body Composition Measurements

Aliquots of the carcass homogenate were analyzed for the determination of lipid, protein, and water content [28]. Water content was determined by the difference in weight of the homogenate before and after drying at 70 °C in a vacuum oven. Lipid content was determined gravimetrically after extraction in chloroform/methanol and evaporation to constant weight by a rotating evaporator (Heidolph, Kelheim, Germany) by the Folch method [29]. Protein content was determined by the Biuret method after extraction in sodium dodecyl sulfate ± NaOH as previously described [30, 31].

### Serum and Tissue Inflammatory Parameters

The serum levels of tumour necrosis factor-α (TNF-α), interleukin-6 (IL-6), interleukin-1β (IL-1β), monocyte chemoattractant protein-1 (MCP-1), and the TNF-α skeletal muscle levels were measured using commercially available ELISA kits (Thermo Scientific, Rockford (TNF-α, IL-6, IL-1β) and BioVendor, Brno, Czechia (MCP-1) as previously performed [32].

### Skeletal Muscle Oxidative Stress and Antioxidant/Detoxyfing Evaluation

ROS levels were measured as previously reported [33]. An aliquot of tissue homogenate was diluted in 100 mM potassium phosphate buffer (pH 7.4) and incubated in a final concentration of 5 mM dichlorofluorescein diacetate (Sigma-Aldrich) in dimethyl sulfoxide for 15 min at 37 °C. The dye-loaded samples were centrifuged at 12,500 × g per 10 min at 4 °C. The pellet was resuspended in 5 mL of 100 mM potassium phosphate buffer (pH 7.4) at 4 °C, and incubated for 60 min at 37 °C. The fluorescence measurements were performed at 488 nm for excitation and 525 nm for emission wavelengths. ROS were quantified using a dichlorofluorescein standard curve in dimethyl sulfoxide (0–1 mM). Catalase (CAT) activity was determined based on the decomposition of H_2_O_2_ at 25 °C [34]. The dithionitrobenzoic acid (DTNB)-GSSG reductase recycling assay was used to measure reduced (GSH) and oxidized (GSSG) glutathione concentrations, with the GSH/GSSG ratio used as an oxidative stress marker. The detoxifying enzymatic activities of NAD(P)H quinone dehydrogenase (NQO1) and glutathione S-transferase (GST) were evaluated spectrophotometrically in cytoplasmic extracts [35–37].

### Mitochondrial Preparation and Analysis

Mitochondrial isolation from skeletal muscle was performed as previously described [37, 38]. Briefly, after removal, limb leg muscle aliquots were freed of excess fat and connective tissue, finely minced, and washed in a medium containing 100 mM KCl, 50 mM Tris-HCl, pH 7.5, 5 mM MgCl_2_, 1 mM EDTA, 5 mM EGTA, 0.1% (w/v) fatty acid-free bovine serum albumin (BSA). Tissue fragments were homogenized with the above medium (1: 8, w/v) in a Potter Elvehjem homogenizer (Heidolph, Kelheim, Germany) set at 500 rpm (4 strokes/min) and filtered through sterile gauze and finally centrifuged (3,000 × g, 10 min, 4 °C). The resulting supernatant was discarded, and the pellet was resuspended and centrifuged at 500 × g for 10 min. The supernatant was centrifuged (3,000 × g, 10 min, 4 °C) and the pellet, containing the mitochondrial fraction, was washed once and resuspended in suspension medium [37]. The protein content of the mitochondrial suspension was determined by the Hartree method using BSA as the protein standard [39]. Mitochondrial oxygen consumption was estimated by a Clark type electrode (Yellow Springs Instruments, Yellow Springs, OH, USA) maintained in a water-jacketed chamber at 30 °C. Skeletal muscle mitochondria (0.5 mg protein) were incubated in a medium containing 80 mM KCl, 50 mM HEPES, 1 mM EGTA, 5 mM KH_2_PO_4_ (pH 7.0), and 0.1% (w/v) fatty-acid-free BSA, as previously described. The substrates used for skeletal muscle respiration were 10 mM succinate + 3.75 μM rotenone or 40 μM palmitoyl-carnitine + 2.5 mM-malate for the determination of fatty acid oxidation rate. State 3 measurements were performed in the presence of 0.6 mM ADP. The ratio between state 3 and 4, called the respiratory control ratio, was calculated according to Estabrook [40]. The addition of ADP after the substrate to the mitochondrial incubation allows to the ATP synthase to function and to electron transport chain to accelerate (“state 3ADP”). When the ATP/ADP ratio approaches equilibrium proton re-entry through the ATP synthase stops and respiration slows (‘state 4’). The degree of coupling was determined in mitochondria as previously reported by applying Equation by Cairns et al. [41]: Degree of coupling = $\sqrt{1}-\frac{\left(\text{Jo}\right)\text{sh}}{\left(\text{Jo}\right)\text{unc}}$ , where (Jo)sh represents the oxygen consumption rate in the presence of oligomycin that inhibits ATP synthase, and (Jo)unc is the uncoupled rate of oxygen consumption induced by carbonyl cyanide 4-(trifluoromethoxy)phenylhydrazone (FCCP), which dissipates the trans-mitochondrial proton gradient. (Jo)sh and (Jo)unc were measured as above using succinate (10 mmol/L) + rotenone (3.75 μmol/L) in the presence of oligomycin (2 μg/mL) or FCCP (1 μmol/L). In control experiments, we assessed the purity of mitochondrial preparation by checking that a possible contamination by other ATPase-containing membranes was below 10%, whereas the quality of mitochondrial preparation was assessed by adding cytochrome c (3 nmol/mg protein) and evaluating an enhancement in state 3 respiration rate ≤10%, as previously indicated [42]. Rate of mitochondrial H_2_O_2_ release was assayed by following the linear increase in fluorescence (ex 312 nm and em 420 nm) due to the oxidation of homovanillic acid in the presence of horseradish peroxidase [43] Superoxide dismutase (SOD) specific activity was measured in a medium containing 0.1 mM EDTA, 2 mM KCN, 50 m KH_2_PO_4_, pH 7.8, 20 mM cytochrome c, 5 m xanthine, and 0.01 U of xanthine oxidase. Enzyme activity was measured spectrophotometrically (550 nm) at 25 °C, by monitoring the decrease in the reduction rate of cytochrome c by superoxide radicals, generated by the xanthine–xanthine oxidase system. One unit of SOD activity is defined as the concentration of enzyme that inhibits cytochrome c reduction by 50% in the presence of xanthine and xanthine oxidase [44].

### Statistical Analysis

All data are presented as means ± SEM. Two-way ANOVA considering the factor diet (CD or HFD) and age (1, 3, 6, 12, or 24 weeks) followed by Tukey’s *post hoc* test was used to evaluate differences between the groups for all the parameters analysed. Differences were considered statistically significant at p < 0.05. Spearman correlation analysis was carried out between body composition, serum/tissue inflammatory parameters, and muscle antioxidant/detoxifying parameters for every pair of Y data set (correlation matrix), two-tailed *p* value (confidence interval 95%), considering the trend of each parameter at time points 6, 12 and 24 weeks. The results were displayed as a heatmap generated based on the correlation coefficients. All analyses were performed using GraphPad Prism (GraphPad Software, San Diego, CA, United States).

## Results

### Body Composition

**FIGURE 1:**
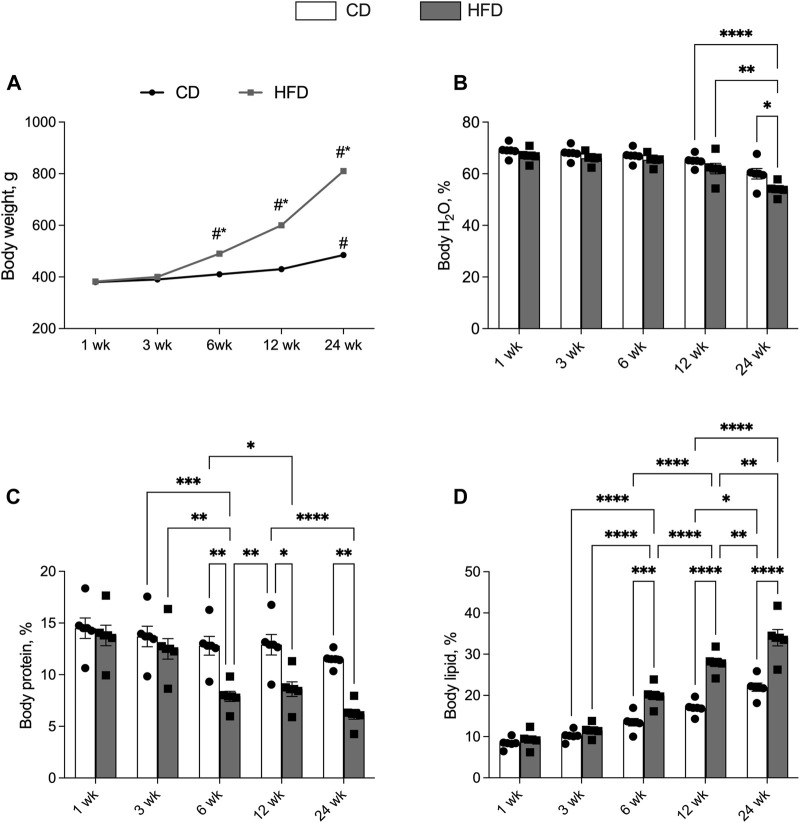
Effects of high-fat diet and age on body composition. All the parameters were measured throughout the experimental period from 1 to 24 weeks (wk). **(A)** Body weight; percentages of **(B)** body water, **(C)** body protein and **(D)** body lipid. Data are indicated as means ± SEM from n = 6 animals/group. Data were compared by two-way ANOVA considering the factor diet (CD or HFD) and age (1, 3, 6, 12, or 24 weeks) followed by Tukey’s *post hoc* test. *p < 0.05; **p < 0.01; ***p < 0.001; ****p < 0.0001; # significantly different compared to previous time point (p < 0.05).

The effects of HFD and aging on body composition were evaluated at 1, 3, 6, 12 and 24 weeks of dietary treatment. Through the experimental period it was observed an increase in body weight in the HFD-fed rats compared with the control group (CD) fed the standard diet (Figure 1A). The percentage of body water and body protein progressively decreases with age both in HFD and CD groups. The reduction in body protein is more relevant in HFD compared to the control group from week 6 (Figure 1C). In contrast, the percentage of body lipids increases progressively with age, with a higher increment in HFD compared to the CD group, starting from about 6 weeks of treatment (Figure 1D).

### Serum Inflammatory Parameters

**FIGURE 2:**
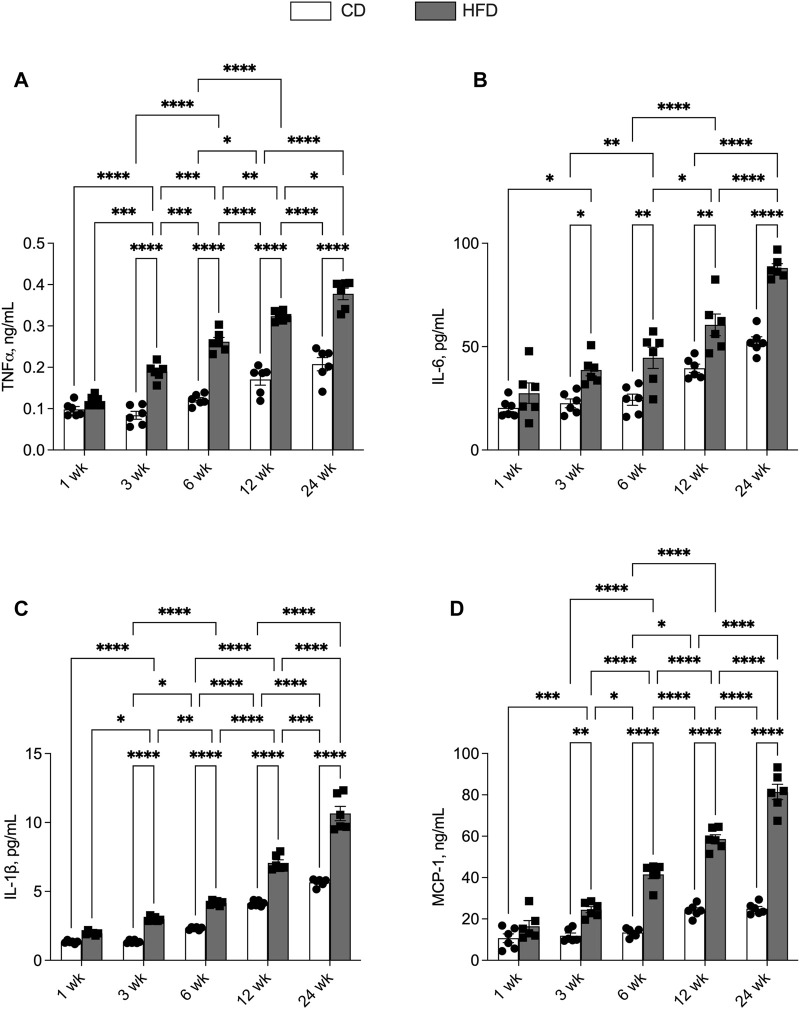
Effects of high-fat diet and age on serum inflammatory parameters. All the parameters were measured throughout the experimental period (1–24 weeks): **(A)** Tumor necrosis factor-α (TNF- α); **(B)** Interleukin-6 (IL-6); **(C)** Interleukin-1β (IL-1β) and **(D)** monocyte chemoattractant protein-1 (MCP-1) levels are shown. Data are indicated as means ± SEM from n = 6 animals/group. Data were compared by two-way ANOVA considering the factor diet (CD or HFD) and age (1, 3, 6, 12, or 24 weeks) followed by Tukey’s *post hoc* test. *p < 0.05; **p < 0.01; ***p < 0.001; ****p < 0.0001.

We then evaluated the effects of HFD and aging on serum levels of pro-inflammatory cytokines associated with obesity-related diseases. CD and HFD groups showed progressively increasing levels of TNF-α, IL-6, IL-1β and MCP-1 with age. The levels of all the analysed cytokines were significantly higher in HFD than CD group, at each time point (Figures 2A–D). The data relating to markers of the lipid profile (NEFA, leptin, adiponectin, triglycerides, cholesterol), glucose metabolism (fasting blood glucose insulin levels and HOMA index) and protein catabolism (urea levels) are reported in Supplementary Figure S1. The alterations of these metabolic markers observed with ageing are exacerbated by the long term HFD feeding (Supplementary Figure S1).

### Skeletal Muscle Inflammatory and Redox Status Parameters

**FIGURE 3:**
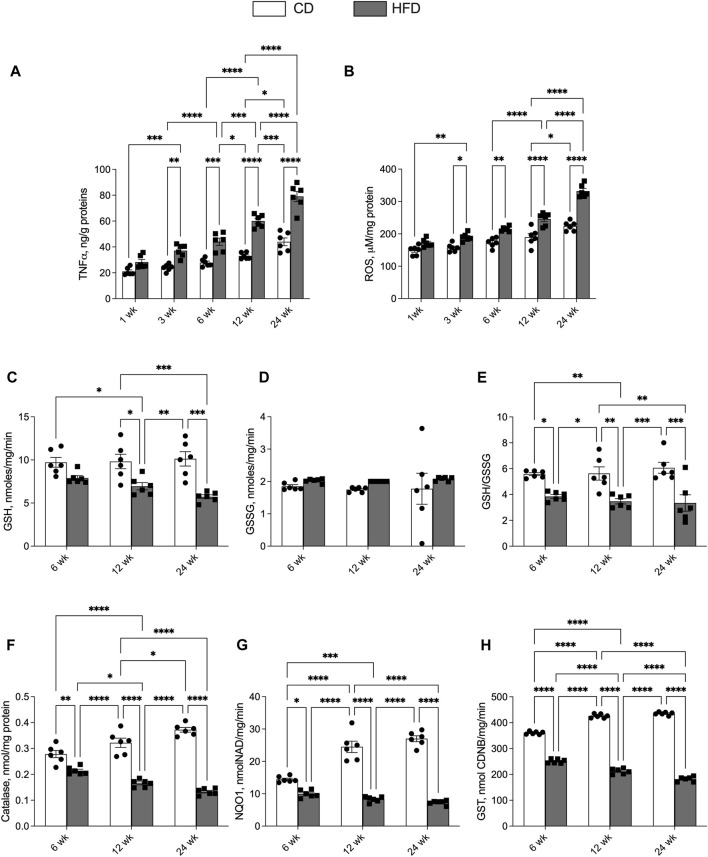
Effects of high-fat diet and age on skeletal muscle inflammatory and oxidative stress parameters. All the parameters were measured throughout the experimental period (1–24 weeks): **(A)** Tumor necrosis factor-α (TNF- α) and **(B)** Reactive oxygen species (ROS) content is shown. Reduced (GSH) and oxidized (GSSG) glutathione content **(C,D)** and ratio GSH/GSSG **(E)** is reported. The enzymatic activities of catalase **(F)**, NAD(P)H quinone oxidoreductase (NQO1) **(G)** and glutathione transferase (GST) **(H)** are also reported. Data are indicated as means ± SEM from n = 6 animals/group. Data were compared by two-way ANOVA considering the factor diet (CD or HFD) and age (1, 3, 6, 12, or 24 weeks) followed by Tukey’s *post hoc* test. *p < 0.05; **p < 0.01; ***p < 0.001; ****p < 0.0001.

We analysed inflammatory and redox status parameters in skeletal muscle to investigate the effects of HFD and aging on inflammation and ROS content in muscle tissue. In HFD rats it was observed a progressive increase in muscle levels of TNF-α and ROS content compared with the CD group at each time point, although the difference was significant starting from the third week of treatment (Figures 3A,B). In the CD group a significant increase in muscle levels of TNF-α and ROS content appeared only after 12 weeks of treatment, although there was an upward trend in previous time points (Figures 3A,B).

GSH content in the CD group does not change with age, while it displays a downward trend in HFD group. Moreover, GSH content is lower in HFD compared to CD group at each time point, with statistically significant differences at 12 and 24 weeks (Figure 3C). The GSSG content did not change with age for both groups (Figure 3D). Thus, the GSH/GSSG ratio was lower in the HFD group compared with CD at each time point, showed a downward trend with age in the HFD group, and did not change in CD group (Figure 3E). Catalase activity displayed an opposite trend in the two groups. An upward trend was observed in the CD group, with statistically significant differences between weeks 12 and 24, while a downward trend was observed in the HFD group, with values lower than in the CD group at each time point (Figure 3F). A similar trend was observed for NQO1 and GST activity levels (Figures 3G,H).

### Skeletal Muscle Mitochondrial Oxidative Capacities

**FIGURE 4:**
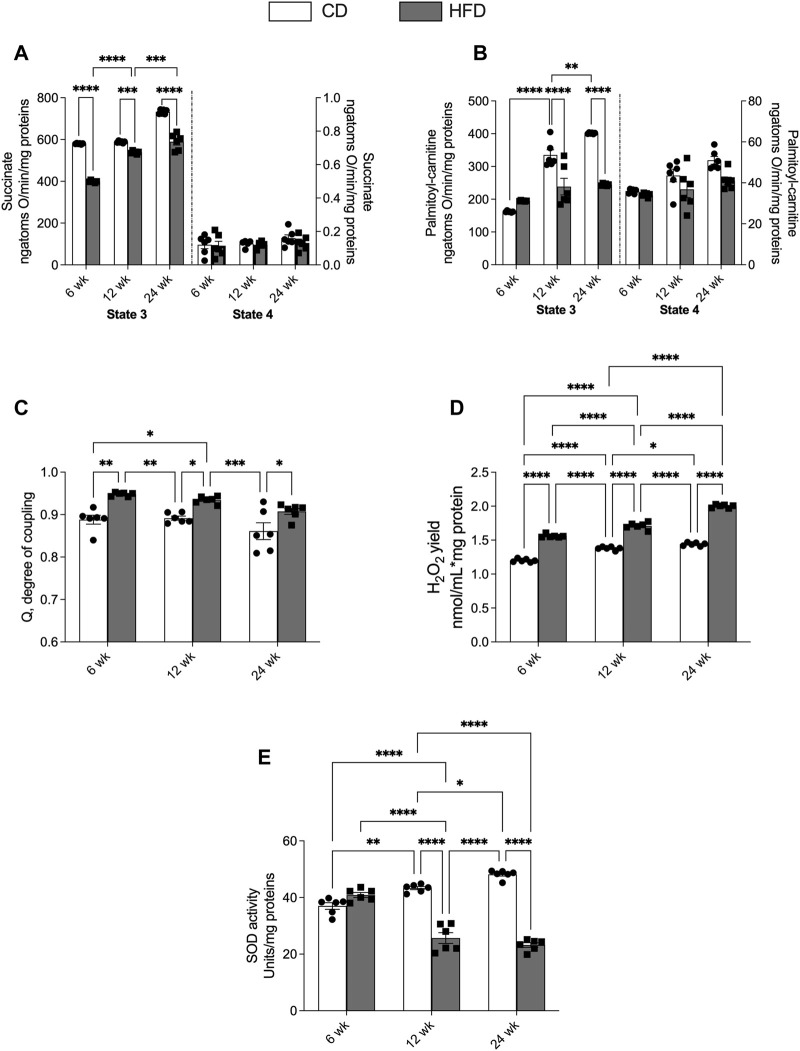
Effects of high-fat diet and age on skeletal muscle mitochondrial oxidative capacity and H_2_O_2_ release. All the parameters were measured throughout the experimental period (6–24 weeks): Mitochondrial respiration rates measured in the presence of **(A)** succinate or **(B)** palmitoyl carnitine as substrates; **(C)** degree of coupling (Q); **(D)** hydrogen peroxide yield (H_2_O_2_); **(E)** superoxide dismutase (SOD) activity. Data are indicated as means ± SEM from n = 6 animals/group. Data were compared by two-way ANOVA considering the factor diet (CD or HFD) and age (1, 3, 6, 12, or 24 weeks) followed by Tukey’s *post hoc* test. *p < 0.05; **p < 0.01; ***p < 0.001; ****p < 0.0001.

Since mitochondria play a crucial role in managing cellular energy and regulating oxidative and inflammatory state, we investigated the functionality of these organelles in the skeletal muscle. Mitochondrial oxidative respiratory activity, using succinate as a substrate in the absence of ADP (state 4), did not show significant changes with age in both CD and HFD groups (Figure 4A). With succinate as substrate, but in the presence of ADP (state 3), we observed a progressive increase in mitochondrial respiratory capacity in both groups of rats with age (Figure 4A). Compared with the CD, the HFD group showed significantly lower state 3 oxygen consumption values at each time point (Figure 4A). When palmitoyl carnitine was used as substrate in the absence or presence of ADP (state 4 or state 3), we found the same trend observed with succinate as substrate (Figure 4B). Skeletal muscle mitochondrial energetic efficiency, measured as the degree of coupling (q) after the addition of the oligomycin and FCCP (data not shown), is significantly higher in the HFD group compared to CD group at each time point (Figure 4C). The hydrogen peroxide (H_2_O_2_) yield increased progressively in both CD and HFD groups from week 6–24. The values of the HFD group at each time point were significantly higher compared to CD group (Figure 4D). In the control group, during aging, we observed a constant antioxidant activity of the SOD, while in the HFD group, SOD activity levels at 12 and 24 weeks were significantly reduced compared to those at 6 weeks, and compared to the control group (Figure 4E).

### Correlations Between Body Composition, Serum/Tissue Inflammatory Parameters, and Muscle Antioxidant/Detoxifying Parameters

**FIGURE 5:**
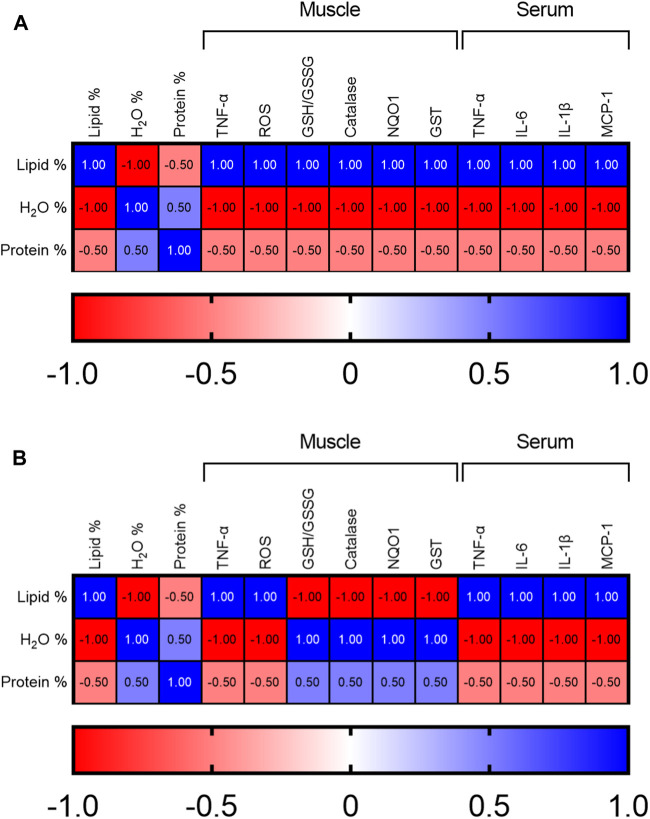
Heatmaps showing Spearman’s correlation analysis between body composition, serum/muscle pro-inflammatory parameters, and muscle antioxidant/detoxifying parameters over time considering weeks 6, 12 and 24 in **(A)** control and **(B)** high-fat diet group.

The relationship between changes in body composition and levels of pro-inflammatory and antioxidant/detoxifying parameters in muscle and serum, considering the sequence of weeks 6, 12 and 24, has been analyzed in both CD and HFD groups. Spearman correlation analysis indicated a negative correlation between the percentage of body lipids and percentage of water and protein, in both groups with advancing age (Figures 5A,B). A positive correlation was observed between lipid percentage and inflammatory cytokines (TNF-a, IL-6, IL-1b and MCP-1) in serum and muscle of both groups (Figures 5A,B). The same positive correlation was also detected between lipid percentage and muscle ROS levels in both groups. Interestingly, it was observed a negative correlation between protein and water percentage and all inflammatory cytokines in serum and muscle of both groups (Figures 5A,B). Noteworthy, the correlation between body lipid percentage and the muscle antioxidant/detoxifying parameters (GSH/GSSG ratio, catalase activity, NQO1 and GST), was opposite in the CD and HFD groups: in the CD group the correlation was positive (Figure 5A), while in the HFD group was negative (Figure 5B). Instead, the correlation between H_2_O and body protein percentage and these parameters was negative in the CD group and positive in the HFD group.

## Discussion

Ageing and obesity shared several metabolic alterations such as changes in body composition, glucose and lipid metabolism, chronic low-grade inflammation, insulin resistance, mitochondrial dysfunctions and oxidative stress. These factors contribute to increased protein catabolism and reduced protein synthesis [45, 46], which combined with altered body composition can lead to sarcopenia. The sarcopenia, a common geriatric syndrome [14], is characterized by the loss of muscle mass and bone tissue, exposing the elderly to walking impairments and increased risk of fractures, which is further exacerbated by the sedentary lifestyle often associated with this stage of life [47]. These alterations can be worsened by obesity, leading to the development of sarcopenic obesity (SO) in some individuals.

Here, we investigated the effects of aging and high-fat diet (HFD) on body composition, inflammation, and oxidative stress using a rodent model. Our results showed that the physiological age-related changes in body composition are anticipated and exacerbated by HFD consumption. Indeed, in the CD group we observed a slight downward trend for body protein percentage and non-significant upward trend for body lipid percentage with age, while the scenario changes in the HFD group in which, starting from 6 weeks of treatment, we detected a significant increase in body weight and body lipid percentage, and a significant decrease in body protein percentage [11]. The increase in body weight and body lipid percentage occurs simultaneously with the increase in the serum levels of inflammatory cytokines typically released by unhealthy adipose tissue [48]. With age, inflammatory cytokines, particularly TNF-α, lead to increased protein catabolism and simultaneously to reduced protein synthesis [49]. This, in addition to a persistent inflammatory condition and an increase in ROS occurring in the muscle itself, leads to a significant reduction in lean mass [50]. In fact, our results show a positive trend for TNF-α and ROS levels in the muscle of the CD group with age. In the HFD-fed group, a significant and much more pronounced increase of these parameters is observed as early as 3 weeks of treatment. These results, together with the changes in body composition, highlight the negative impact of HFD able to anticipate and worsen the metabolic events that in the CD group appear later in age, and are less severe.

ROS are known to have beneficial or harmful effects depending on their dose. In fact, they are an example of a molecule with a hormone-like response, with a signaling function at physiological low doses, but able to induce cellular damage at higher doses [51]. For this reason, the term “eustress” is used to indicate the physiological effects elicited by ROS, while the term oxidative “distress” indicates the excessive ROS levels, contributing to a range of pathological events. Mitochondria are among the main sources of intracellular ROS production. In line with this, the increase in oxidative stress and ROS production characterizing aging is accompanied by the progressive decline in mitochondrial capacity [52]. However, the biphasic model of age-associated mitochondrial functions has been proposed [21, 53]. We investigated the mitochondrial functions at different time points in both HFD and CD group. In CD group we observed that the increased mitochondrial capacity with age is associated with mild ROS production that is able to stimulate physiological antioxidant responses limiting the aging-associated damages. In HFD group we observed impaired mitochondrial functions, associated with higher ROS production that may not be counteracted by antioxidant responses, in line with previous observations [54–56]. A key antioxidant defence cellular system is the pathway associated to Nrf2, an oxidative stress sensor that controls the transcriptional induction of genes involved in cellular protection and detoxification processes, such as NQO1 and GST [57]. We investigated the antioxidant responses in our model evaluating the GSG/GSSG ratio, and the activity of catalase, as well as of two enzymes, NQO1 and GST, which are under the control of Nrf2. Our data showed that HFD hinders the physiological detoxification attempts of the cells. Indeed, the activity of catalase, NQO1 and GST gradually increases with age in the CD group, while progressively decreasing in the HFD group. We concluded that in the HFD group the antioxidant defences fail their job because of the additional inflammation and oxidative stress due to the diet itself. It is well known that HFD is related to decreased animals’ activity [58]. Thus, cannot be excluded that the reduced physical activity may contribute, at least in part, to the impaired mitochondrial functions in the skeletal muscle of HFD rats. Moreover, mitochondrial dysfunction in skeletal muscle affects not only the overall muscle fibers but also muscle progenitor cells, such as satellite cells that are critical for muscle repair and regeneration, particularly under conditions of stress or injury. The satellite cells rely on mitochondrial activity for energy production and ROS signaling to regulate their proliferation, differentiation, and fusion into mature muscle fibers [59]. Therefore, the increased oxidative stress and mitochondrial dysfunction associated with HFD may lead to impaired muscle regeneration over time [60]. The impaired capacity of muscle stem cells to repair muscle tissue could contribute to the progression of sarcopenia and the exacerbation of sarcopenic obesity [61, 62].

Altogether, our data provide novel longitudinal evidence that HFD consumption anticipates the age-associated decline in skeletal muscle mitochondrial functions and exacerbates the metabolic and inflammatory alterations associated with aging. This comprehensive approach emphasizes the temporal relationship between diet, aging, and sarcopenia. By integrating the biphasic model of mitochondrial dysfunction with this longitudinal design, we offer new insights into the mechanisms linking chronic inflammation, mitochondrial decline, and the development of sarcopenic obesity. These findings have important implications for understanding the pathophysiology of sarcopenic obesity and may contribute to the development of targeted interventions aimed at mitigating age-related muscle loss and improving metabolic health in the elderly.

## Summary Table

### What Is Known About This Subject

- Aging alter body composition, inflammation and oxidative stress, obesity state exacerbates these alterations
- Mitochondrial capacity declines with aging, but may follow a biphasic trajectory from young adult to middle age.
- Sarcopenia involves loss of muscle mass and mitochondrial dysfunction with aging, obesity can worsen it.

### What This Work Adds

- Longitudinal data show that HFD anticipates and worsens age-related metabolic and inflammatory alterations.
- HFD increases muscle TNF-α/ROS early and is linked to impaired mitochondrial function versus controls.
- Antioxidant defenses rise with age in controls but progressively fall with HFD, suggesting failed compensation.

## Concluding Statement

This work represents an advance in biomedical science because it defines when HFD shifts aging from adaptive to damaging redox/mitochondrial changes, accelerating sarcopenia onset.

## Data Availability

The raw data supporting the conclusions of this article will be made available by the authors, without undue reservation.
