# Supplementary material for: High-Fat Diet Anticipates Age-Related Sarcopenia Through Increased Oxidative Stress and Inflammation
Source: Br J Biomed Sci. 2026 Feb 26;83:15743. doi: 10.3389/bjbs.2026.15743 (PMC12979236; doi:10.3389/bjbs.2026.15743)
Supplement: Supplementary file 1 [file Supplementaryfile1.docx]

Supplementary materials

1. Materials and methods

Serum concentrations of triglycerides (TG) and cholesterol, and non-esterified fatty acids (NEFAs) were measured by colorimetric enzymatic method using commercial kits (SGM Italia, Italy and Randox Laboratories ltd., United Kingdom). Commercially available ELISA kits were used to determine adiponectin and leptin (B-Bridge International, Mountain View, CA, U.S.A.), insulin (cat.no. EZRMI-13K; Millipore, Germany) and Urea (MyBioSource, San Diego, CA, U.S.A.) levels. Glucose levels were determined by glucometer (Contour next, Ascensia, Italy). HOmeostasis Model Assessment (HOMA) of IR was calculated [HOMA=fasting glucose (mg/dL) × fasting insulin (μg/ L)/14.1] ^1^.


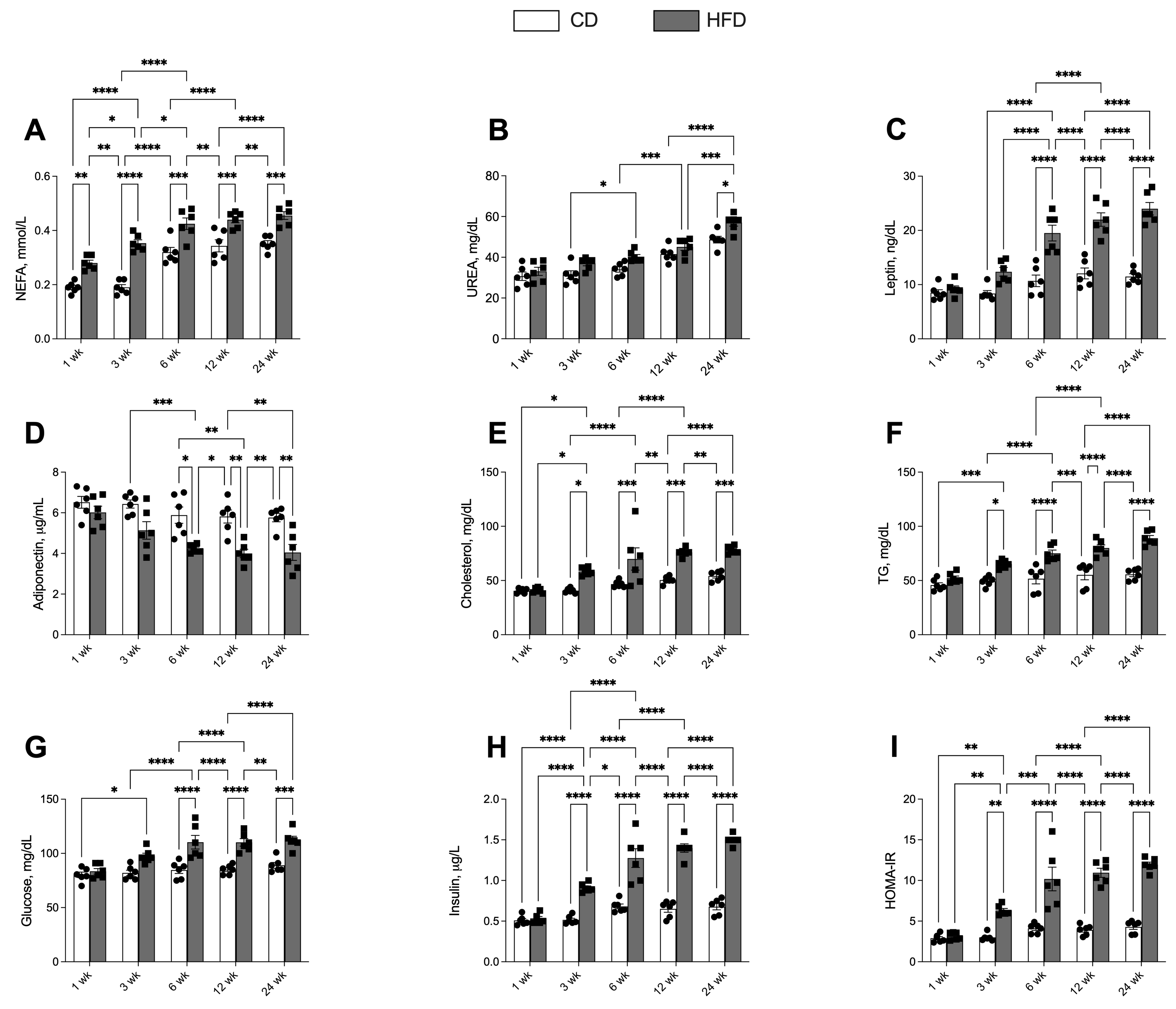


Figure legend

**Supplementary Figure 1.** Effects of high-fat diet and age on serum metabolic parameters. All the parameters were measured throughout the experimental period (1–24 weeks): (A) Non-esterified fatty acids (NEFA); (B) Urea; (C) Leptin; (D) Adiponectin; (E) Cholesterol; (F) Triglycerides (TG); (G) Glucose; (H) Insulin; (I) HOMA-IR levels are shown. Data are indicated as means ± SEM from n = 6 animals/group. Data were compared by two-way ANOVA considering the factor diet (CD or HFD) and age (1, 3, 6, 12, or 24 weeks) followed by Tukey’s post hoc test. * p<0.05; ** p<0.01; ***p<0.001; ****p<0.0001.

References

(1) Cacho, J.; Sevillano, J.; de Castro, J.; Herrera, E.; Ramos, M. P. Validation of Simple Indexes to Assess Insulin Sensitivity during Pregnancy in Wistar and Sprague-Dawley Rats. *Am J Physiol Endocrinol Metab* **2008**, *295* (5), E1269-1276. https://doi.org/10.1152/ajpendo.90207.2008.
